# Bloodstream infections in adult patients with malignancy, epidemiology, microbiology, and risk factors associated with mortality and multi-drug resistance

**DOI:** 10.1186/s12879-021-06243-z

**Published:** 2021-07-02

**Authors:** Ali Amanati, Sarvin Sajedianfard, Somayeh Khajeh, Shabnam Ghasempour, Salma Mehrangiz, Samane Nematolahi, Zahra Shahhosein

**Affiliations:** 1 Professor Alborzi Clinical Microbiology Research Center, Shiraz University of Medical Sciences, Shiraz, Iran; 2 Shiraz University of Medical Sciences, Shiraz, 7193711351 Iran

**Keywords:** Bloodstream infection, Carbapenem-resistant isolates, Extended-Spectrum Beta-lactamase producing pathogens, Multidrug-resistant gram-negative infection, Mortality, Cancer

## Abstract

**Background:**

This study aimed to investigate the epidemiology, microbiology, and risk factors associated with mortality and multi-drug resistance bacterial bloodstream infections (BSIs) among adult cancer patients in Shiraz, Iran. We also report a four-year trend of antimicrobial resistance patterns of BSIs.

**Methods:**

We conducted a retrospective study at a referral oncology hospital from July 2015 to August 2019, which included all adults with confirmed BSI.

**Results:**

2393 blood cultures tested during the four-year study period; 414 positive cultures were included. The mean age of our patients was 47.57 ± 17.46 years old. Central Line-Associated BSI (CLABSI) was more common in solid tumors than patients with hematological malignancies. Gram-negative (GN) bacteria were more detected (63.3%, 262) than gram-positive bacteria (36.7%, 152). *Escherichia coli* was the most common gram-negative organism (123/262, 47%), followed by *Pseudomonas spp.* (82/262, 31%) and *Klebsiella pneumoniae* (38/262, 14.5%). *Coagulase-negative staphylococci* (CoNS) was the most frequently isolated pathogen among gram-positive bacteria (83/152, 54.6%). *Acinetobacter spp.*, *Pseudomonas spp*., *E. coli*, and *K. pneumoniae* were the most common Extended-Spectrum Beta-Lactamase (ESBL) producers (100, 96.2, 66.7%, and 60.7, respectively). *Acinetobacter* spp., *Pseudomonas* spp., *Enterobacter* spp., *E. coli*, and *K. pneumoniae* were the most common carbapenem-resistant (CR) isolates (77.8, 70.7, 33.3, 24.4, and 13.2%, respectively). Out of 257 *Enterobacterales* and non-fermenter gram-negative BSIs, 39.3% (101/257) were carbapenem-resistant. Although the incidence of multi-drug resistance (MDR) gram-negative BSI increased annually during 2015–2018, the mortality rate of gram-negative BSI remains unchanged at about 20% (*p*-value = 0.55); however, the mortality rate was significantly greater (35.4%) in those with resistant gram-positive BSI (p-value = 0.001). The overall mortality rate was 21.5%. Early (7-day mortality) and late mortality rate (30-day mortality) were 10 and 3.4%, respectively.

**Conclusions:**

The emergence of MDR gram-negative BSI is a significant healthcare problem in oncology centers. The high proportion of the most frequently isolated pathogens were CR and ESBL-producing *Enterobacterales* and *Pseudomonas spp.* We have few effective choices against MDRGN BSI, especially in high-risk cancer patients, which necessitate newer treatment options.

**Supplementary Information:**

The online version contains supplementary material available at 10.1186/s12879-021-06243-z.

## Background

Bacterial bloodstream infection (BSI) is one of the most common complications of chemotherapy-induced neutropenia in patients with hematologic malignancies and solid organ tumors [1–3], which is associated with high mortality and morbidity [4–8]. Bacterial BSIs account for the etiologic cause of approximately 20 to 30% of all febrile neutropenic episodes in adult patients with malignancy [9, 10]. While proper diagnosis and treatment are essential to decrease BSI-associated complications, inappropriate empiric antimicrobial therapy increases mortality [11]. BSI’s reported crude mortality rates to reach as high as 34 to 50%, especially in MDR gram-negative BSI [9, 12]. The antimicrobial stewardship program (ASP) helps to reduce the overuse of antibiotics and control the increased antimicrobial resistance. Surveillance of antimicrobial resistance is one of ASP’s critical aspects and guides clinicians for appropriate empiric antimicrobial therapy [13, 14].

This study aimed to determine the current epidemiology of bacterial BSI and its changes during the different study years in a large cohort of patients with solid organ and hematological malignancy. We also assessed BSI attributed mortality risk factors and MDR gram-negative BSI predictors.

## Methods

### Setting and data collection

This study performed at Amir oncology hospital, an educational 100-bed inpatient center. The adult units consist of four inpatient wards and an autologous hematopoietic stem cell transplantation ward. Since 2015 our institution has carried out a blood culture surveillance program using an automated blood culture system (BD BACTEC™). Hospital Information System (HIS) and microbiology department records used for data collection. Patients followed up 30 days after BSI by infectious disease specialist consultant (AA) and chief infection control unit staff (MS).

### Study population and design

In this retrospective single-center study, we analyzed all consecutive episodes of BSI occurring in adult patients with hematological malignancies and solid organ tumors from July 2015 to August 2019. Each patient ≥18 years of age with positive blood culture was considered to BSI when he/she had clinical signs and symptoms of bacteremia. Patients with a fungal infection, contaminant result, or a mix of more than two organisms, excluded from this study. Finally, 414 patients recruited, and data analyzed for age, gender, underlying diseases, presence of central venous catheters, leucocyte count, neutropenia, etiologic microorganisms, susceptibility testing, and outcome.

### Definitions

Patient with a recognized bacterial pathogen, which not included on the commensal list, identified from one or more blood specimens obtained by culture and at least one of the following signs or symptoms: fever (> 38 °C), chills, or hypotension and not be related to an infection at another site considered as true BSI [15].

Febrile neutropenia defined as temperature > 38.5 °C or two consecutive temperature > 38 °C for 2 h and an absolute neutrophil count< 0.5 X 10^9^ cell/L or expected to fall below < 0.5 X 10^9^ cell/L. Imipenem, meropenem, cefepime, piperacillin-tazobactam, and colistin are available on our hospital formulary. We use piperacillin-tazobactam and carbapenem as the first-line agents for patients with febrile neutropenia.

Methicillin-resistant coagulase-negative *staphylococci* (MRCoNS) defined as **cefoxitin**-resistant strains. *Enterobacteriaceae* family, *P. aeruginosa*, and *A. baumannii* isolates resistant to ceftazidime or cefotaxime ar**e** considered extended-spectrum beta-lactamase (ESBL) producers [16]. Phenotypic confirmation of ESBL production carried out by using the double-disk synergy test [17]. Carbapenem-resistant *Enterobacterales* (CREs), carbapenem-resistant *Pseudomonas spp.*, and carbapenem-resistant *Acinetobacter spp.* isolates defined as Enterobacterales that test intermediate or resistant to one or more carbapenems using the CLSI current breakpoints. However, not all isolates tested against all carbapenems [16]. MDR defined as the strain non-susceptible to at least one agent in ≥3 classes of antibiotics, including carbapenems, combinations of beta-lactams plus beta-lactamase inhibitors, cephalosporins, aminoglycosides, and fluoroquinolones [18].

### Microbiological methods

Blood cultures obtained by physicians based on clinical suspicion of bacteremia. BACTEC™ FX Automated Blood Culture Systems used for detection of BSI. Time-to-Detection (TTD) defined as the time between the placement of each blood culture bottle in the incubation cabinet and the detection of growth. Based on the result of the gram-stain, the subculture routinely was performed, and differentiation tests such as catalase, oxidase, coagulase, bacitracin, optochin, and CAMP test were applied. In our center, the susceptibility testing is done based on the disc diffusion method (Kirby-Bauer method) according to the Clinical and Laboratory Standards Institute (CLSI) guidelines [17] using commercial antibiotic discs (MAST Group Ltd.; UK).

### Statistical analysis

In this study, we used a univariate logistic regression model to examine the critical factors that may be influencing the patient’s survival status and possible predictors of MDR gram-negative BSI. All variables in the univariate analysis (*P* ≤ 0.25) and variables with clinical significance entered into a multivariable model. The independent variables of sex, age, malignancy, ESR, CRP, leukopenia, neutropenia, febrile neutropenia, year, and *Enterobacterales* separately entered a logistic regression model. We reported odds ratio values and also the confidence interval of the odds ratio for each variable. A *p*-value < 0.05 was considered statistically significant. The analysis was done by SPSS version 25 (IBM Corp., Armonk, NY, USA).

## Results

### Demographics and epidemiology

**Fig. 1:**
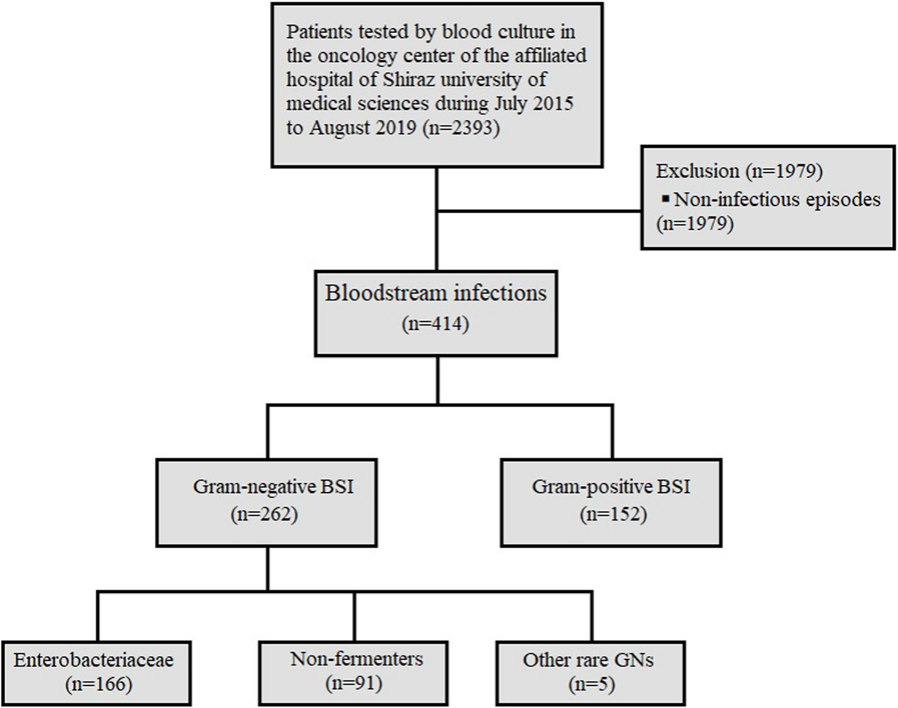
Flow chart of study population screened and enrolled associated with bloodstream infections caused by gram-negative and gram-positive bacteria in patients treated at the Amir oncology hospital between July 2015 to August 2019

In this study, four-hundred, fourteen positive blood cultures were analyzed (Fig. 1). Two hundred twenty-three patients were male (53.9%). The mean age was 47.57 ± 17.46 years old. Hematologic malignancies (212, 51.4%), solid tumors (167, 40.3%), and non-malignant disorders (20, 4.8%), including aplastic anemia, were the most common underlying diseases, respectively.

### Clinical and laboratory features

**Fig. 2:**
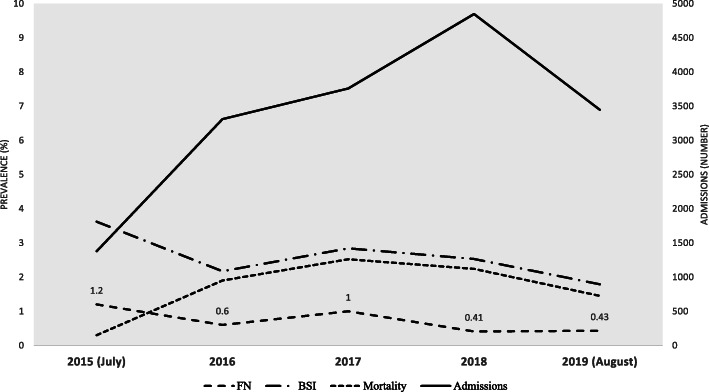
Incidence of febrile neutropenia, bloodstream infections, mortality rate/10000 cases, and hospitalized adults with cancer during 2015–2019. FN: febrile neutropenia episodes; BSI: bloodstream infection

The mean of white blood cell (WBC), erythrocyte sedimentation rate (ESR), and C-reactive protein (CRP) were (6.94 ± 11.28) X 10^9^ /L, (71.68 ± 37.26) mm/hour, (82.59 ± 41.44) mg/dl, respectively. ESR and CRP were not statistically different between two malignancy types (*P* = 0.6646; 95% CI: − 7.07 to 11.07 and *P* = 0.0663, 95% CI: − 19.64 to 0.64, respectively). Neutropenia was more prominent in those with hematologic malignancies (117, 69.6%) compare with patients with solid organ tumors (38, 22.6%); *P* < 0.001. Febrile neutropenia also was significantly higher in those with hematologic malignancies (83, 74.8%; P < 0.001). Overall, 63.5% of positive culture was primary BSI, and 36.5% was CLABSI. CLABSI was more common in those with solid tumors; the difference was not significant *p* = 0.165. The most reported TTD was less than 12 h (188/414, 50%), while 30.3% detected during 12–24 h, 11.7% between 24 and 36 h, and only 8% diagnosed in more than 36 h by BACTEC™ Systems. The rate of admission, BSI, febrile neutropenia, and mortality during the study years are shown in Fig. 2. The summary characteristics of the patients with solid tumors and hematological malignancies could be found in Table S1 in the supplement file.

### Microbiology

**Fig. 3:**
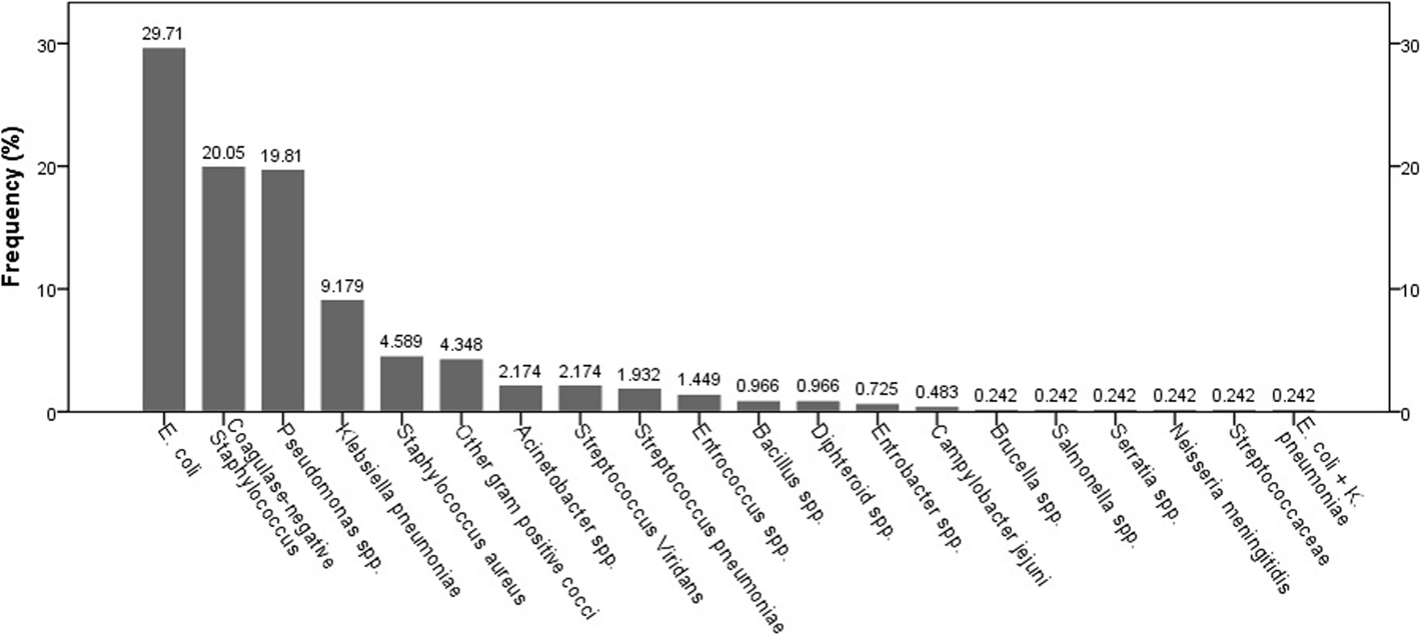
The percentage frequency distribution of different gram-negative and gram-positive bacteria isolated from blood cultures

262 (63.3%) and 152 (36.7%) gram-negative and gram-positive pathogens organisms isolated from blood cultures. *Escherichia coli* was the most common gram-negative organism (123/262, 47%), followed by *Pseudomonas* spp. (82/262, 31%) and *K. pneumoniae* (38/262, 14.5%). Coagulase-negative *staphylococci* (CoNS) was the most frequently isolated pathogen among gram-positive bacteria (83/152, 54.6%). The incidence of gram-positive and gram-negative bacteria isolated from blood culture is shown in Fig. 3. *Acinetobacter spp.*, *Pseudomonas spp*., *E. coli*, and *K. pneumoniae* were the most common ESBL producers (100, 96.2, 66.7%, and 60.7, respectively).

*Acinetobacter* spp., *Pseudomonas* spp., *Enterobacter* spp., *E. coli*, and *K. pneumoniae* were the most common carbapenem-resistant isolates (77.8, 70.7, 33.3, 24.4, and 13.2%, respectively).

Out of 257 *Enterobacterales* and non-fermenter gram-negative BSIs, 39.3% (101/257) were carbapenem-resistant. The incidence of CRE and carbapenem-resistant non-fermenter BSIs increased annually between 2015 and 2018 (*p* < 0.001).

**Fig. 4:**
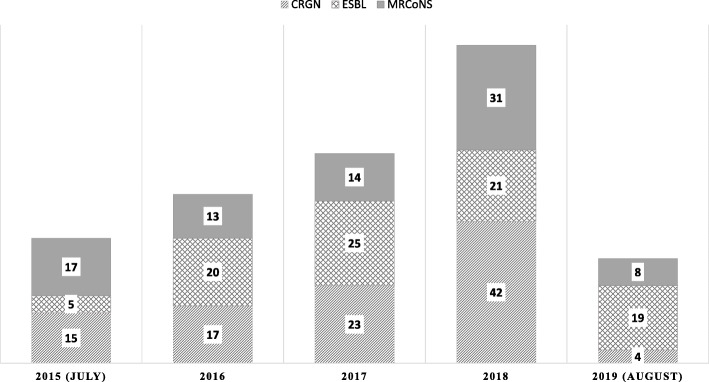
The annual frequency of ESBL and CRE-associated BSI, in addition to MRCoNS, associated BSI (labels represent case numbers). CRGN includes carbapenem resistance *Enterobacterales* and non-fermenter spp.

The frequency of isolated organisms, MRCoNS, ESBL, and CRGN associated BSI, is shown in Fig. 4. As shown, there are emerging ESBL and CRGN BSIs during different study years.

**Fig. 5:**
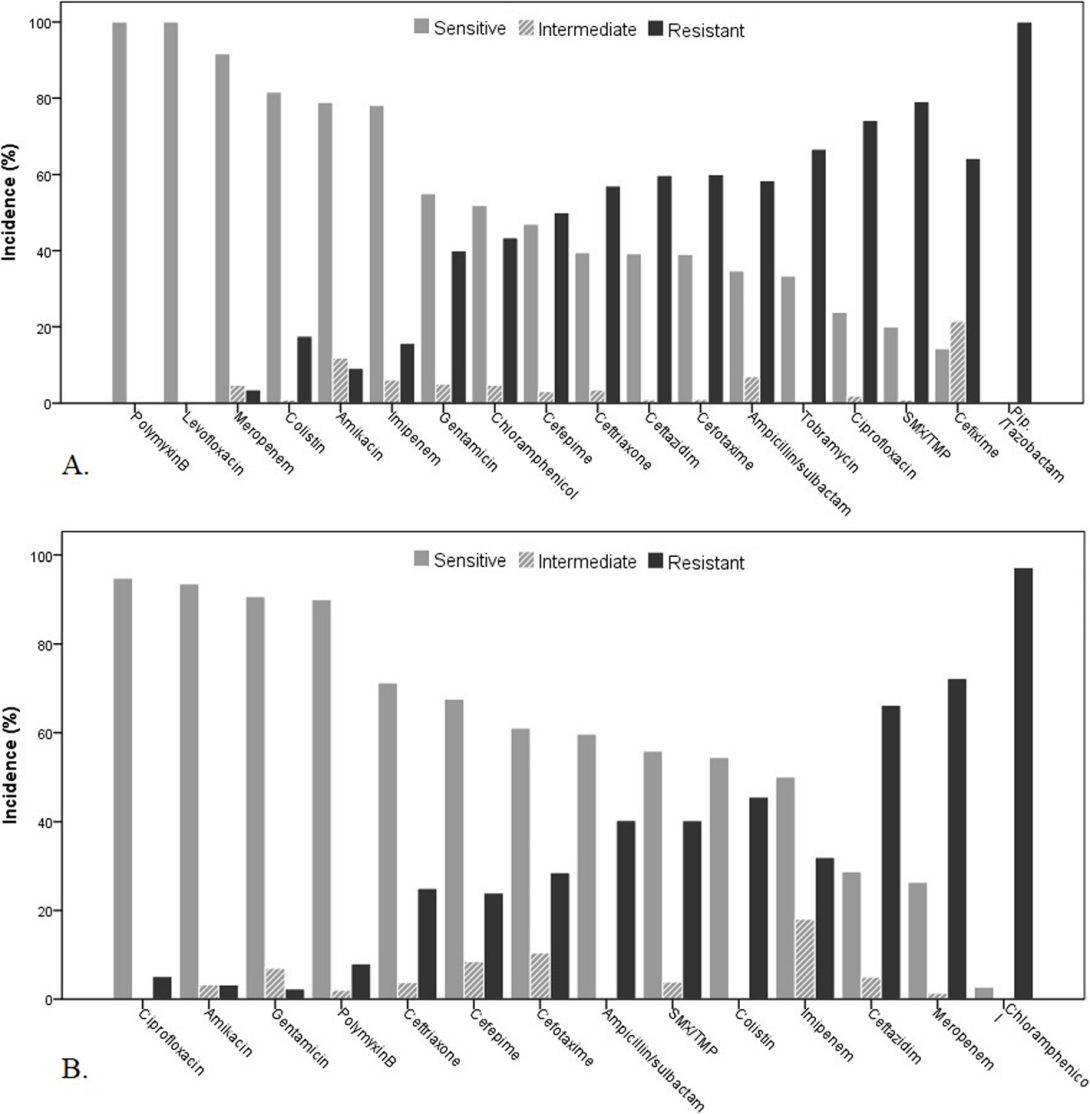
Antimicrobial susceptibility results of 123 *E. coli* (A) and 81 *Pseudomonas* spp. (B) isolates recovered from blood cultures during 2015–2019

**Fig. 6:**
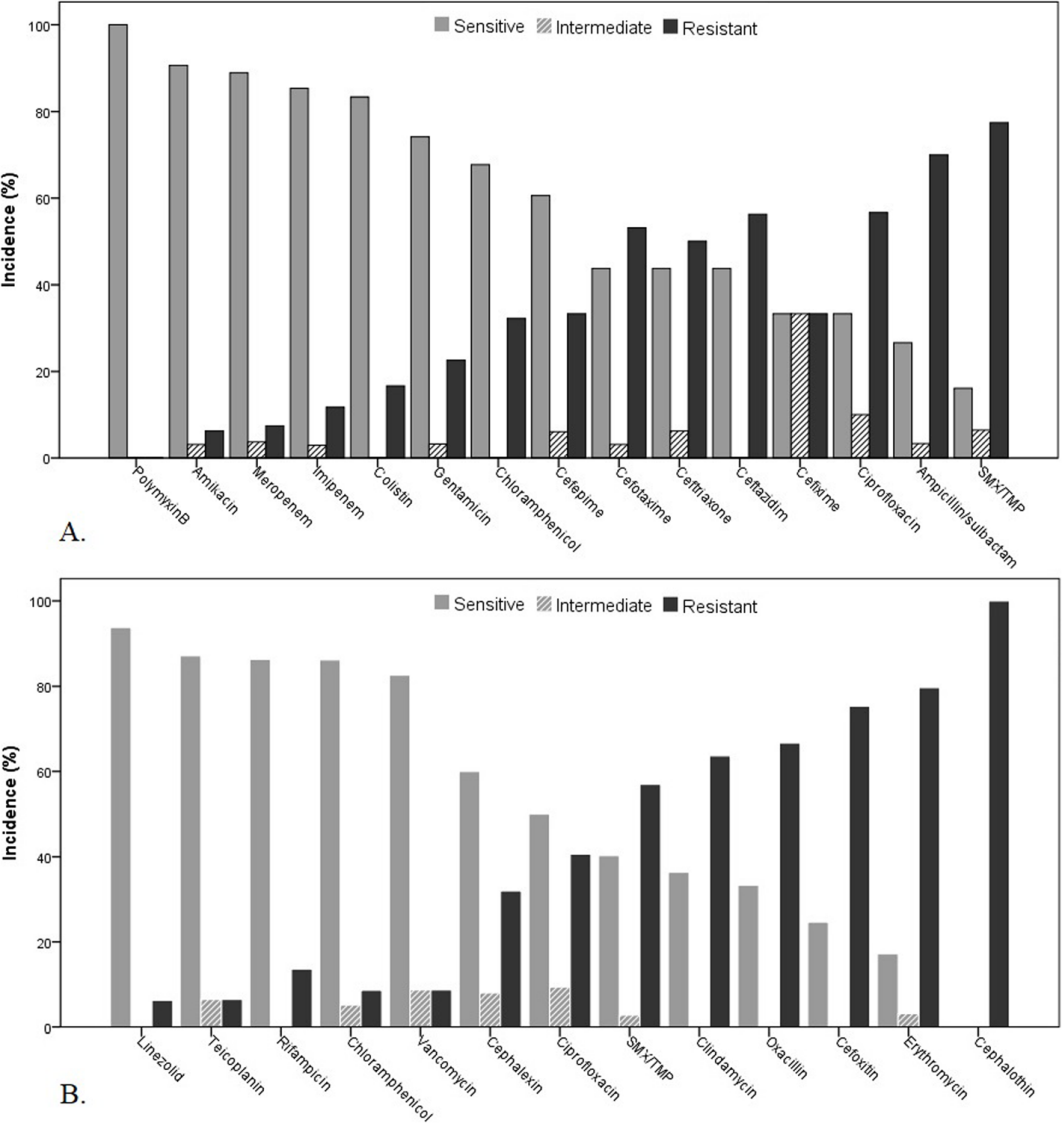
Antimicrobial susceptibility results of 38 *K. pneumonia* (A) and of 80 *coagulase-negative staphylococci* (CoNS) isolates (B) recovered from blood cultures during 2015–2019

Antibiotic susceptibility results for the most common isolated bacteria are shown in Figs. 5 and 6. All *E. coli* were sensitive to polymyxin b. The sensitivity of *E. coli* for meropenem, colistin, amikacin, and imipenem were 92, 82.79, and 78%, respectively. All *E. coli* was resistant to piperacillin-tazobactam. More than 90% of *Pseudomonas* spp. were sensitive to ciprofloxacin, amikacin, gentamicin, and polymyxin-b, while 97% were chloramphenicol- resistant. All *K. pneumoniae* was sensitive to polymyxin-b, and 91% were susceptible to amikacin. More than 80% of *K. pneumoniae* was sensitive to meropenem, imipenem, and colistin. Trimethoprim/sulfamethoxazole and ampicillin-sulbactam found less sensitive agents. Most *coagulase-negative staphylococci* (94%) were susceptible to linezolid, and more than 80% of them were susceptible to teicoplanin, rifampicin, chloramphenicol, and vancomycin.

Among the *Enterobacteriaceae* family, 91 (64.1%) isolates were ESBL-producer, and 36 (28.8%) were CRE. CR detected in 71.6% *P. aeruginosa* and 87.5% of *Acinetobacter spp.*

Overall, in those with gram-negative BSI, 49.3% found to be non-susceptible to at least one agent in ≥3 classes of antibiotics, including carbapenems (imipenem or meropenem), combinations of beta-lactams plus beta-lactamase inhibitors (piperacillin-tazobactam or ampicillin-sulbactam), cephalosporins (3rd or 4th generation cephalosporins), aminoglycosides (amikacin or gentamicin), or fluoroquinolones (ciprofloxacin or levofloxacin). Although the MDR gram-negative (MDRGN) BSI cases increased from July 2015 to 2018, the difference was not significant (*p* = 0.588). Table 1 represents the incidence of MRCoNS, ESBL producing organisms, CRE and *Acinetobacter spp.* and *Pseudomonas spp.* in different study years.

**Table 1:** MDRGN, ESBL, and CRGN associated BSIs frequency among most common gram-negative bacteria as well as MRSA and MRCoNS among gram-positive associated BSIs during 2015–2019

		2015 n (%)	2016 n (%)	2017 n (%)	2018 n (%)	2019 n (%)	p-value
MDR		20 (40)	36 (50)	53 (49.5)	66 (53.75)	29 (46.8)	0.588
	*E. coli*	12 (60)	22 (61.1)	26 (49.1)	13 (19.7)	18 (62.1)	
	*K. pneumoniae*	3 (15)	3 (8.3)	4 (7.5)	9 (13.6)	5 (17.2)	
	*Acinetobacter spp.*	2 (10)	3 (8.3)	2 (3.8)	0 (0)	0 (0)	
	*Pseudomonas spp.*	1 (5)	6 (16.7)	18 (34)	43 (65.2)	6 (20.7)	
ESBL		5 (15.2)	31 (46.3) *	43 (43) *	66 (55.5)	28 (45.9)	**0.002**
	*E. coli*	5 (100)	18 (75)	20 (60.6)	14 (82.4)	13 (50)	
	*K. pneumoniae*	0 (0)	3 (15)	4 (16)	7 (33.3)	6 (31.6)	
	*Acinetobacter spp.*	0 (0)	2 (100)	3 (100)	0 (0)	0 (0)	
	*Pseudomonas spp.*	0 (0)	6 (100)	15 (88.2)	45 (97.8)	9 (100)	
CRGN		15 (14.9)	17 (16.8)	23 (22.8)	42 (41.6)	4 (4)	**< 0.0001**
	*E. coli*	11 (84.6)	11 (40.7)	5 (13.9)	3 (14.3)	0 (0)	
	*K. pneumoniae*	2 (66.7)	2 (40)	0 (0)	1 (7.1)	0 (0)	
	*Acinetobacter spp.*	1 (33.3)	3 (100)	3 (100)	0 (0)	0 (0)	
	*Pseudomonas spp.*	0 (0)	1 (16.7)	15 (75)	38 (82.6)	4 (44.4)	
MRSA		0 (0)	3 (50)	1 (25)	0 (0)	0 (0)	**0.038**
MR-CONS		13 (86.7)	7 (53.8)	13 (92.9)	22 (78.6)	5 (62.5)	0.112

MDRGN: multidrug-resistant gram-negative bacteria; ESBL: extended-spectrum beta-lactamases (ESBL)-producing gram-negative bacteria; CRE: carbapenem-resistant *Enterobacterales*; CRGN: carbapenem-resistant gram-negative bacteria; MRCONS: methicillin-resistant *coagulase-negative Staphylococci*; MRSA: methicillin-resistant *Staphylococcus aureus*. *P*-values marked with bold indicate statistically significant *p*-values

Among tested ESBL-producer gram-negative isolates, susceptibility to polymyxin-b, amikacin, colistin, imipenem, and meropenem, were 95, 87, 70, 65.4, and 54.5%, respectively. Polymyxin-b, amikacin, and colistin found the most active agent against CRE clinical isolates (87.5, 75, and 43.2%, respectively).

Sixty cases detected as *MRCoNS* (76.9%). Among MRCoNS, 94% were susceptible to linezolid, and more than 80% were susceptible to teicoplanin, chloramphenicol, and rifampin, while vancomycin was less sensitive (74%). All of the isolated enterococci were VRE.

### Clinical influence of drug-resistance BSI and predictors of mortality

Based on the obtained results, third-generation cephalosporin-resistant *E. Coli* and *P. aeruginosa*, carbapenem-resistant *P. aeruginosa*, and polymyxin-resistant *K. pneumoniae* BSIs associated with increased mortality; however, the difference was not statistically significant (*p*-value: 0.719, > 0.999, 0.521, and 0.467; respectively).

Based on results of univariate logistic regression analysis of variables investigated for survival in cancer patients with BSI, none of them associated with a greater risk of mortality (Table 2). Our results revealed that the odds of mortality decreased annually during 2015–2018 (Table 2).

**Table 2:** Univariate logistic regression analysis of variables investigated for prediction of mortality in cancer patients with bloodstream infection

	Alive (*n* = 324, 78.5%)	Dead (*n* = 89, 21.5%)	OR (95% CI)	*p*-value
Sex				
Female	136 (45.8)	37 (45.7)	1.005 (0.613,1.645)	0.986
Male*	161 (54.2)	44 (54.3)		
Age				
< 60	209 (70.4)	52(64.2)	1.325 (0.789,2.224)	0.288
> 60*	88 (29.6)	29(35.8)		
Malignancy type				
Solid organ tumor*	131 (44.1)	36 (44.4)	1.014 (0.618,1.662)	0.957
Hematologic malignancy	166 (55.9)	45 (55.6)		
ESR	73.58 (36.10)	63.77 (36.88)	1.008 (0.999,1.017)	0.091
CRP	82.12 (43.12)	88.36 (28.62)	0.996 (0.989,1.004)	0.332
WBC count				
< 4000/μl*	169 (57.3)	49 (60.5)	1.142 (0.691,1.885)	0.605
> 4000/μl	126 (42.7)	32 (39.5)		
Neutropenia				
Yes (< 1500/μl)	121 (41.3)	33 (41.3)	1.002 (0.606,1.656)	0.994
No (> 1500/μl) *	172 (58.7)	47 (58.8)		
Febrile neutropenia				
No	212 (71.4)	60 (74.1)	0.873 (0.500,1.524)	0.633
Yes*	85 (28.6)	21 (25.9)		
Year				
2015	36 (12.1)	6 (7.4)	1.773 (0.612,5.132)	0.291
2016	52 (17.5)	11 (13.6)	1.397 (0.569,3.427)	0.466
2017	81 (27.3)	22 (27.2)	1.088 (0.500,2.368)	0.832
2018	84 (28.3)	29 (35.8)	0.856 (0.405,1.810)	0.684
2019*	44 (14.8)	13 (16)		
Enterobacterales				
No*	70 (36.1)	18 (38.3)	1.100 (0.570,2.121)	0.777
Yes	124 (63.9)	29 (61.7)		

* Reference

Besides, univariate logistic regression analysis showed that only WBC count < 4000/μl (OR: 1.753; 1.159–2.651) and non-fermenter gram-negative BSI (OR: 3.120; 1.481–6.575) were associated with greater risk for MDR gram-negative BSI (*p*-value 0.008 and 0.003, respectively). Furthermore, we found that the odds of MDR gram-negative BSI increased annually during 2016–2018 (Table 3).

**Table 3:** Univariate logistic regression analysis of variables investigated for prediction of MDR gram-negative infection in cancer patients with bloodstream infection

	Non-MDR (*n* = 19,50.4%)	MDR (*n* = 188, 49.6%)	OR (95% CI)	*p*-value
Sex				
Female	78 (40.8)	95 (50.5)	1.48 (0.986, 2.221)	0.059
Male*	113 (59.2)	93 (49.5)		
Age				
< 60	125 (65.4)	136 (72.3)	1.381 (0.892,2.138)	0.148
> 60*	66 (34.6)	52 (27.7)		
Malignancy				
Solid organ tumor	84 (44)	83 (44.1)	1.007 (0.671,1.511)	0.973
Hematologic malignancy*	107 (56)	105 (55.9)		
ESR	67.96 (34.76)	75.40 (37.68)	1.006 (0.999,1.013)	0.101
CRP	78.60 (37.28)	88.05 (43.48)	1.006 (1.000,1.012)	0.065
WBC count				
< 4000/μl	97 (51.3)	122 (64.9)	1.753 (1.159,2.651)	**0.008**
> 4000/μl*	92 (48.7)	66 (35.1)		
Neutropenia				
Yes (< 1500/μl)	69 (36.7)	86 (46.2)	1.483 (0.981,2.243)	0.062
No (> 1500/μl) *	119 (63.3)	100 (53.8)		
Febrile neutropenia				
No*	47 (24.6)	59 (31.4)	1.401 (0.893,2.200)	0.142
Yes	144 (75.4)	129 (68.6)		
Year				
2015	24 (12.6)	18 (9.6)	0.960 (0.429,2.146)	0.921
2016	32 (16.8)	31 (16.5)	1.240 (0.604,2.546)	0.558
2017	51 (26.7)	52 (27.7)	1.305 (0.681,2.501)	0.422
2018	52 (27.2)	62 (33)	1.526 (0.805,2.894)	0.195
2019*	32 (16.8)	25 (13.3)		
*Enterobacterales*				
No **	44 (81.5)	110 (58.8)	3.120 (1.481,6.575)	**0.003**
Yes*	10 (18.5)	78 (41.5)		

* Reference

** non-fermenter gram-negative BSI. *P*-values marked with bold indicate statistically significant *p*-values

Given the emergence of CRGN bacteria isolated in blood culture of patients with BSI during 2015–2018 (as we shown in Fig. 4) and the high proportion of CR isolates in non-fermenters (more than 70%) and also in *Enterobacterales* (about 25%), we investigated the odds of different possible risk factors for carbapenem-resistant BSI by logistic regression model (Table 4). Accordingly, we found that age < 60-year-old, solid organ neoplasms, non-fermenter gram-negative BSI, third-generation cephalosporine resistant gram-negative isolates, and polymyxin-resistance infections were significantly associated with carbapenem-resistant BSI based on univariate logistic regression analysis.

**Table 4:** Logistic regression analysis of factors associated with carbapenem-resistant gram-negative bacterial bloodstream infection in cancer patients

	n (%)	n (%)	Univariate	*p*-value
Age				
< 60	78 (83)	54(56.8)	3.701 (1.887,7.262)	**< 0.0001**
> 60*	16 (17)	41(43.2)		
Malignancy type				
Solid organ	55 (58.5)	35 (36.8)	2.418 (1.347,4.339)	**0.003**
Hematologic malignancy*	39 (41.5)	60 (63.2)		
*Enterobacterales*				
Yes*	33 (35.1)	81 (85.3)	10.695 (5.269,21.709)	**< 0.0001**
No	61 (64.9)	14 (14.7)		
4th GCRGN				
Resistance	42 (44.7)	31 (32.6)	1.667 (0.923,3.011)	0.090
Sensitive*	52 (55.3)	64 (67.4)		
3rd GCRGN				
Resistance	88 (93.6)	57 (60)	9.778 (3.884,24.615)	**< 0.0001**
Sensitive*	6 (6.4)	38 (40)		
Aminoglycoside-resistant				
Resistance	31 (33)	21 (22.1)	1.734 (0.907,3.314)	0.096
Sensitive*	63 (67)	74 (77.9)		
Polymyxin-resistance				
Resistance	50 (53.2)	6 (6.3)	16.856 (6.713,42.323)	**< 0.0001**
Sensitive*	44 (46.8)	89 (93.7)		

* Reference. *P*-values marked with bold indicate statistically significant *p*-values

**Fig. 7:**
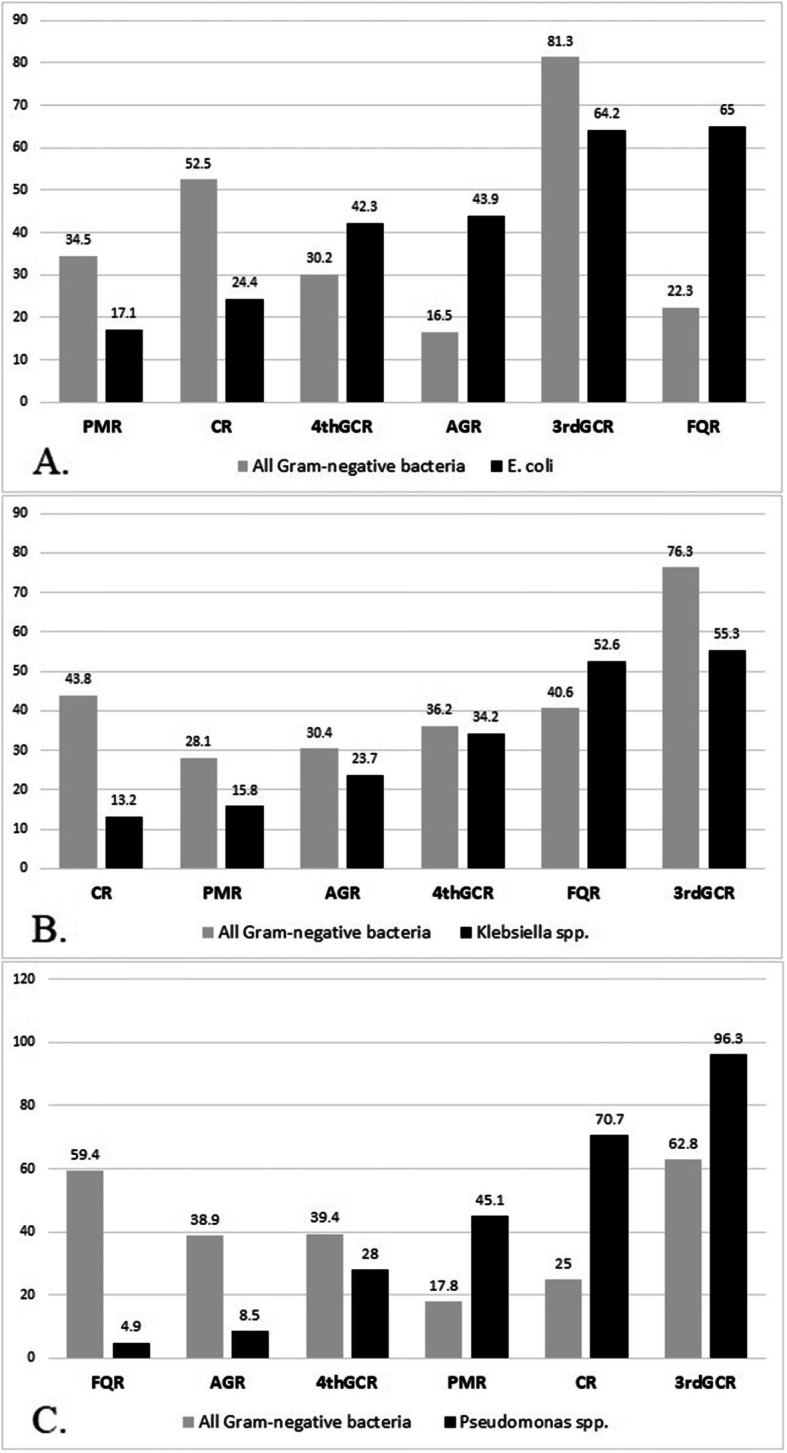
Comparative analysis for antibiotic resistance rate between *E. coli*, *K*. *pneumoniae*, and *Pseudomonas* spp. with all gram-negative isolates (A, B, and C, respectively)

PMR: polymyxin-resistant; CR: carbapenem-resistant; 3rdGCR: third-generation cephalosporine-resistant gram-negative isolates; 4thGCR: fourth-generation cephalosporine-resistant gram-negative isolates; AGR: aminoglycoside- resistant; FQR: fluoroquinolone-resistant.

As illustrated in Fig. 7, based on both univariate and multivariate logistic regression analysis, *Pseudomonas*-associated BSI and polymyxin-resistance BSI were significantly associated with carbapenem-resistant BSI. Among tested antibiotics, only fluoroquinolones (including ciprofloxacin and levofloxacin) and aminoglycosides have acceptable sensitivity (resistance rate lesser than 10%) for *Pseudomonas* associated BSI. For *E. coli*-associated BSIs, polymyxins were the most active drug, while polymyxins and carbapenems were the best choices for *K. pneumoniae*-associated BSI (resistance rate < 15%).

Detail information regarding susceptibility profile of MDR, CR, and ESBL-producer *E. coli*, *Pseudomonas spp.*, and *K. pneumoniae* isolates against different antimicrobial classes summarized in Table 5.

**Table 5:** The susceptibility profile of carbapenem-resistant and ESBL-producer *E. coli*, *Pseudomonas spp.*, and *K. pneumoniae* isolates against different antimicrobial classes

Sensitivity rate (n, %)	4th GC	Ciprofloxacin	AGs	BL/BLI	Polymyxins	Carbapenems
Carbapenem-resistant						
*E. coli*	16 (53.3%)	8 (26.7%)	9 (30%)	11 (36.7%)	18 (69.2%)	–
*Pseudomonas* spp.	35 (60.3%)	54 (93.1%)	51 (78.9%)	47 (81%)	6 (66.6%)	–
*K. pneumoniae*	2 (40%)	1 (20%)	2 (25%)	2 (40%)	5 (62.5%)	–
ESBL						
*E. coli*	20 (28.6%)	17 (24.3%)	38 (54.3%)	21 (30%)	59 (84.3%)	51 (72.9%)
*Pseudomonas* spp.	52 (69.3%)	71 (94.7%)	70 (93.3%)	53 (70.7%)	41 (54.7%)	20 (26.7%)
*K. pneumoniae*	7 (35%)	6 (30%)	16 (80%)	1 (5%)	6 (75%)	17 (85%)
MDR						
*E. coli*	42 (46.2%)	19 (20.9%)	40 (44%)	27 (29.7%)	71 (78%)	62 (68.1%)
*Pseudomonas* spp.	51 (68.9%)	70 (94.6%)	67 (90.5%)	49 (66.2%)	37 (50%)	18 (24.3%)
*K. pneumoniae*	11 (45.8%)	6 (25%)	15 (62.5%)	3 (12.5%)	19 (79.2%)	19 (79.2%)

4^th^ GC: fourth-generation cephalosporins (cefepime); AGs: aminoglycosides (amikacin and gentamicin); BL/BLI: beta-lactamase/beta-lactamase inhibitors combination (piperacillin-tazobactam)

## Discussion

Patients with malignancy are predisposed to developing BSI during their chemotherapy courses. Lots of evidence showed that the epidemiology of nosocomial infections in cancer patients changed over the past decades, with the reemergence of GNB as the predominant causative pathogens. The current study, therefore, conducted to describe the antibiotic-resistant patterns and outcomes of nosocomial infections caused by GNB in adult cancer patients.

Overall, despite the annual increase in the admission rate, no significant increase in the BSI incidence rate and attributed death occurred during the five-year study period (Fig. 2). Like some other reports, our study showed that CLABSI was more common in solid tumors than hematologic malignancies [19]; however, a higher prevalence of CLABSI detected in patients with hematological malignancies in other studies [20]. We found that gram-negative BSI was the most common etiology of BSI in cancer patients (63.3%), which agrees with other reports [21, 22]. Like recent reports, we observed a gradual increase in the incidence of MDRGN and CRGN associated BSIs annually during our surveillance [23, 24].

*E. coli*, *Pseudomonas spp.*, and *K. pneumoniae* were the most common recovered gram-negative isolates in our study, which is in line with previous studies conducted in cancer patients [12, 21, 22, 25]. We found a high proportion of ESBL producers and carbapenem-resistant isolates, predominantly in non-fermenters (96.4 and 82.3%) and the *Enterobacteriaceae* family (64.1 and 28.8%). Although there are scarce reports on the incidence of carbapenem-resistant GNs in our region, obtained results are comparable to available reports [26]; however, compared to Europe, North America, Latin America global surveillance studies, and Asia-Pacific regional surveillance studies, our results showed significant higher-resistance rate [27]. We observed that the incidence of MDR BSI increased from 2015 to 2018.

The overall mortality rate of GNB BSIs among cancer patients in our study was about 20%, which was more significant compared with studies conducted in our region, for example, Calik Basaran et al. (17.0%) [28] and Garcia-Vidal et al. (14.8%) [12]; however, is much lower than some other studies, for example, Al-Otaibi et al. (32.1%) [5], and Yawei Zhang et al. (33.5%) [29].

The mortality rate of carbapenem-resistant *Enterobacterales*, ESBL producing *Enterobacterales*, MDR *Acinetobacter* spp., and MDR *P. aeruginosa* is around 6–7% in the US, and Europe reports [24, 30]. However, in this study, the BSI attributed mortality rate among MDRGN, CRGN, ESBL producer, *Pseudomonas spp.*, *E. coli*, and *K. pneumoniae* were 20.2, 18.8, 19.1, 22, 22.1, and 21.5%, respectively.

We did not observe an association between investigated factors and survival; however, in a study by Chien-Yuan Chen et al. age ≥ 60 years, prior allogeneic transplantation and BSI due to VRE found as independent predictors for mortality [31]. CRGN BSI [26, 32–34], unresolved neutropenia, monotherapy, septic shock [32, 35], and polymicrobial BSI [32] are other risk factors associated with increased mortality in cancer patients with GN BSI.

We identified MDR gram-negative infections more likely to occur in patients with WBC count< 4000 and non-fermenter gram-negative BSI (Table 3). Other studies found that male sex, age ≥ 60, previous antimicrobial use, liver disease, and bacteremia caused by *K. pneumoniae* are associated with increased risk of MDRGN BSI [36].

In agreement with current concerns regarding the efficacy of colistin against carbapenem-resistant pathogens, including CRE [37–40], we found that CR *E. coli* and *K. pneumoniae* isolates did not show acceptable sensitivity to our available antimicrobial choices, including colistin. However, ESBL producer *K. pneumoniae* and ESBL *E. coli* isolates had acceptable sensitivity to carbapenems and aminoglycosides (> 80%) and polymyxins (84.3%), respectively. Like the previous reports, our study reemphasized that carbapenems could still act as the drugs of choice in ESBL associated BSIs in cancer patients [41]. Besides, MDR *E. coli* and *K. pneumoniae* showed a high resistance rate to available antimicrobial agents. Our findings are consistent with previous studies concerning the global emergence of MDRGN pathogens as a significant healthcare burden that could be attributed to the overuse of antibiotics and necessitates the development of new antibiotics for treating CRE, CR *P. aeruginosa*, and CR *Acinetobacter baumannii* [42–44].

We also found that MDR, CR, and ESBL *Pseudomonas spp.* isolates still are sensitive to ciprofloxacin in our setting and could be considered a good treatment choice as recommended by current guidelines [45]. Remarkably, piperacillin-tazobactam, which is frequently preferred as one of the initial antibiotic therapies in our febrile neutropenic patients, may not be an excellent empiric choice against CR *E. coli* and *K. pneumoniae*. Our study showed that carbapenems might be less active against ESBL-producer *Pseudomonas spp.* and to some extent against ESBL-producer *E. coli*. In MDRGN BSI, none of our empiric treatments (carbapenems and piperacillin-tazobactam) could overcome GN bacteremia and need other choices such as colistin (Table 5). Although febrile neutropenia should be considered a medical emergency and a prompt administration of empirical antibiotic therapy is mandatory, increased mortality could be seen due to inappropriate empiric antibiotic therapy in setting with high rates of resistant pathogens [2, 12, 19, 46]. Accordingly, regular epidemiological and microbiological surveillance of BSI should be encouraged strongly in oncology centers.

### Limitation

Our study has some limitations. First, given the retrospective nature of this study, it was difficult to collect some variables (chemotherapeutic protocols, antibiotics treatment before admission, and some clinical and laboratory examination results) in this retrospective study. So, there might be hidden biases in the analysis of the relationship. Second, carbapenemase type and enzyme were not investigated in this study. Finally, our research was conducted with data collected from a single center. Therefore, a prospective multicenter study is needed for further validation.

## Conclusions

In conclusion, the overall case-fatality rate of BSI caused by GNB among cancer patients was 20%. The high proportion of the most frequently isolated pathogens were CR and ESBL-producing *Enterobacterales* and *Pseudomonas spp.* Although fluoroquinolones remain a good choice for CR *P. aeruginosa*, we have few effective choices against CR *E. coli* and *K. pneumoniae*. The new agents such as ceftazidime/avibactam could be helpful in such cases.

## Supplementary Information

**Additional file 1: Table S1.** The summary characteristics of the patients with solid tumors and hematological malignancies.

## Data Availability

The datasets used and analyzed during the current study are available from the corresponding author on reasonable request.

## Abbreviations

BSI: bloodstream infections
CLABSI: Central Line-Associated BSI
ASP: Antimicrobial stewardship program
GN: gram-negative
CR: Carbapenem-resistant
MRCoNS: Methicillin-resistant *coagulase-negative staphylococci*
ESBL: extended-spectrum beta-lactamase
CRE: Carbapenem-resistant *Enterobacterales*
CRGN: Carbapenem-resistant gram-negative
MDRGN: multi-drug resistance gram-negative
