# Supplementary material for: Bloodstream infections in adult patients with malignancy, epidemiology, microbiology, and risk factors associated with mortality and multi-drug resistance
Source: BMC Infect Dis. 2021 Jul 2;21:636. doi: 10.1186/s12879-021-06243-z (PMC8254331; doi:10.1186/s12879-021-06243-z)
Supplement: Supplementary file 1 — Additional file 1: Table S1. The summary characteristics of the patients with solid tumors and hematological malignancies. [file 12879_2021_6243_MOESM1_ESM.docx]

**Supplement**

Table-S1 Statistical analysis of antigram-negative agents against bloodstream infections caused by MDR-gram negative isolates

| Antibiotics | Sensitivity | MDRGNB | | P-Value* |
| --- | --- | --- | --- | --- |
|  |  | Count | % |  |
| Imipenem | Sensitive | 86 | 54.1% | **<0.001** |
|  | Intermediate | 23 | 14.5% |  |
|  | Resistant | 50 | 31.4% |  |
| Meropenem | Sensitive | 68 | 50.0% | **<0.001** |
|  | Intermediate | 6 | 4.4% |  |
|  | Resistant | 62 | 45.6% |  |
| Gentamicin | Sensitive | 77 | 63.6% | 0.109 |
|  | Intermediate | 8 | 6.6% |  |
|  | Resistant | 36 | 29.8% |  |
| Amikacin | Sensitive | 115 | 80.4% | 0.062 |
|  | Intermediate | 12 | 8.4% |  |
|  | Resistant | 16 | 11.2% |  |
| Ampicillin-sulbactam | Sensitive | 54 | 38.3% | 0.034 |
|  | Intermediate | 3 | 2.1% |  |
|  | Resistant | 84 | 59.6% |  |
| Cefotaxime | Sensitive | 47 | 32.2% | **<0.001** |
|  | Intermediate | 6 | 4.1% |  |
|  | Resistant | 93 | 63.7% |  |
| Ceftriaxone | Sensitive | 59 | 36.4% | **<0.001** |
|  | Intermediate | 7 | 4.3% |  |
|  | Resistant | 96 | 59.3% |  |
| Ceftazidime | Sensitive | 16 | 10.7% | **<0.001** |
|  | Intermediate | 5 | 3.4% |  |
|  | Resistant | 128 | 85.9% |  |
| Cefepime | Sensitive | 48 | 34.5% | **<0.001** |
|  | Intermediate | 12 | 8.6% |  |
|  | Resistant | 79 | 56.8% |  |
| Colistin | Sensitive | 104 | 64.2% | **0.001** |
|  | Intermediate | 1 | .6% |  |
|  | Resistant | 57 | 35.2% |  |
| Ciprofloxacin | Sensitive | 71 | 46.4% | **0.048** |
|  | Intermediate | 7 | 4.6% |  |
|  | Resistant | 75 | 49.0% |  |
| Chloramphenicol | Sensitive | 56 | 37.1% | **<0.001** |
|  | Intermediate | 5 | 3.3% |  |
|  | Resistant | 90 | 59.6% |  |
| Trimethoprim/sulfamethoxazole | sensitive | 58 | 36.9% | 0.284 |
|  | intermediate | 3 | 1.9% |  |
|  | resistant | 96 | 61.1% |  |
| Polymyxin-B | Sensitive | 47 | 90.4% | 0.517 |
|  | Intermediate | 1 | 1.9% |  |
|  | Resistant | 4 | 7.7% |  |

*Statistically significant p-values presented by bold numbers
